# A Comparative Study of On-Body Radio-Frequency Links in the 420 MHz–2.4 GHz Range

**DOI:** 10.3390/s18124165

**Published:** 2018-11-27

**Authors:** Arno Thielens, Robin Benarrouch, Stijn Wielandt, Matthew G. Anderson, Ali Moin, Andreia Cathelin, Jan M. Rabaey

**Affiliations:** 1 Berkeley Wireless Research Center, Department of Electrical Engineering and Computer Sciences, University of California Berkeley, Berkeley, CA 94704, USA; robin.benarrouch@berkeley.edu or robin.benarrouch.etu@univ-lille.fr or robin.benarrouch@st.com (R.B.); stijn@berkeley.edu or stijn.wielandt@kuleuven.be (S.W.); matthew_g_anderson@berkeley.edu (M.G.A.); moin@berkeley.edu (A.M.); jan_rabaey@berkeley.edu (J.M.R.); 2 Waves Research Group, IMEC, Department of Information Technology, Ghent University, 9052 Ghent, Belgium; 3 CNRS, Centrale Lille, ISEN, University Valenciennes, UMR 8520—IEMN, University Lille, F-59000 Lille, France; 4 STMicroelectronics, Technology and Design Platforms, 38920 Crolles, France; andreia.cathelin@st.com; 5 DRAMCO, Department of Electrical Engineering (ESAT), Ghent Technology Campus, KU Leuven, 9000 Ghent, Belgium

**Keywords:** body area networks, on-body communication, body-coupled communication, UHF RFID, Bluetooth, RF beam steering, RF repeaters, path loss measurements

## Abstract

While there exists a wide variety of radio frequency (RF) technologies amenable for usage in Wireless Body Area Networks (WBANs), which have been studied separately before, it is currently still unclear how their performance compares in true on-body scenarios. In this paper, a single reference on-body scenario—that is, propagation along the arm—is used to experimentally compare six distinct RF technologies (between 420 MHz and 2.4 GHz) in terms of path loss. To further quantify on-body path loss, measurements for five different on-body scenarios are presented as well. To compensate for the effect of often large path losses, two mitigation strategies to (dynamically) improve on-body links are introduced and experimentally verified: beam steering using a phased array, and usage of on-body RF repeaters. The results of this study can serve as a tool for WBAN designers to aid in the selection of the right RF frequency and technology for their application.

## 1. Introduction

Interest in Wireless Body Area Networks (WBANs) has grown in the last decade because of their potential applications in several wearable applications, predominantly in health applications [1,2]. Previous research of WBANs led to the proposal of a standard for wireless communication (IEEE 802.15.6) [3], while other research focused specifically on different layers of the WBAN stack [4]. 

WBANs are faced with several challenges, including variable coverage due to dynamic aspects of the channels during movement [5], limited energy availability in wearable nodes [6], bandwidth requirements imposed by the applications [7], and desired flexibility in WBAN architecture [8]. These challenges are quite prominent in the physical layer. Propagation in WBANs is not only dependent on the environment, as in conventional wireless networks, but also on static and dynamic interactions with the body. Evidence of this is provided by several studies focussing on the (dynamic) on-body and off-body propagation of radio-frequency (RF) electromagnetic waves [7,9,10,11]. A complicating factor is that WBANs face restrictions on their emitted power due to limits on the available energy in wearable nodes as well as potential exposure risks [12]. To address some of these concerns, other options of wireless communication around the human body beyond RF have been explored such as acoustic [13], optical [14], and electromagnetic millimeter-wave [15] technologies. Given the very demanding constraints in terms of energy efficiency, coverage, robustness and performance imposed on WBANs [16], it is very likely that the optimal network configuration will use a combination of physical propagation mechanisms rather than the homogeneous single-technology solution advocated in the WBAN standard of today. Hence, a fair comparison between the different available technologies and a demonstration of their advantages and drawbacks features would be of very good use.

Even if we restrict the discussion to RF on-body communication only, there still exist different methods in dealing with the aforementioned challenges, each with their own benefits and pitfalls. Passive RF (backscattering) techniques allow for on-body nodes without an energy reservoir, but are limited in readout range [17]. An example of passive on-body RF communication is ultra-high frequency radio-frequency identification (UHF RFID), which operates in a frequency band around 915 MHz [17]. To improve performance, active RF communication using on-body antennas can be employed in multiple WBAN configurations. However, link performance depends on the availability of energy reservoirs in the WBAN nodes and may suffer from signal loss in dynamic scenarios such as movement and lack of line-of-sight [16], although RF beam steering and multi-hop communication using repeaters can be used to mitigate some of these effects [4,5,8,16]. Body coupled communication (BCC) using RF fields can be efficient in situations where on-body RF is not (e.g. through-body links), but is – up to now– limited in range, frequency, and bandwidth [18]. Depending on the requirements imposed for particular sensor nodes on certain positions on the body, different RF methods might be more suitable to establish communication between nodes at a given location and time [16]. However, up to now there is little guidance on how to make those decisions and selections.

To address this need this study presents the following novelties: first, we compare, for the first time, a diverse set of on-body RF technologies (six in total) in a fixed on-body scenario; second, to our knowledge, we are the first paper to compare on-body path loss at 915 MHz in five different on-body scenarios. These results are important because it is expected that UHF RFID at this frequency will gain importance in the future [19]; third, we present a novel capacitive BCC technique; and fourth, we demonstrate a new phased array for on-body beam steering.

A number of papers studied the wireless performance, including path loss, for different on-body configurations at different RF frequencies [20]. However, a considerable number of such studies [21,22,23,24,25] focus on a set of specific on-body links, for example RF communication from head to wrist. Other studies use a systematic approach, where antennas are placed in an on-body scenario, for example on the arm, with different separation distances [26,27,28,29,30,31]. The focus of these studies is to develop a path-loss model rather than characterize the on-body fading in certain scenarios using measurements or simulations. These path loss models can be used to assess the properties of on-body links. Unfortunately, these studies are either done at a single frequency [26,27,30] or a limited number of frequencies under a few scenarios [28,29,31,32,33], and only consider RF antennas, omitting BCC.

Therefore, the first goal of this manuscript is to compare different RF approaches for establishing wireless on-body communication. To this aim, we consider different target RF frequency bands between 0.4–2.4 GHz, where we employ at least one out of two different options: on-body RF communication and BCC. These mechanisms are compared by executing on-body path loss measurements in a reference on-body scenario. Second, we compare 915 MHz propagation in five different on-body scenarios, in order to compare the reference on-body scenario to other on-body locations. Third, we present two novel RF on-body strategies that can help in overcoming some of the previously mentioned challenges in WBANs: on-body repeaters and on-body beam steering.

After a description of the methods and technologies used for the comparison of the different on-body RF mechanisms, the results of our study are presented, discussed and compared to literature, leading to some global conclusions and identification of topics for future research.

## 2. Materials and Methods

In this section the methodology used to compare different RF technologies is described, followed by a detailed overview of the different RF technologies and their specific parameters.

### 2.1. Methodology and Scope

**Figure 1:**
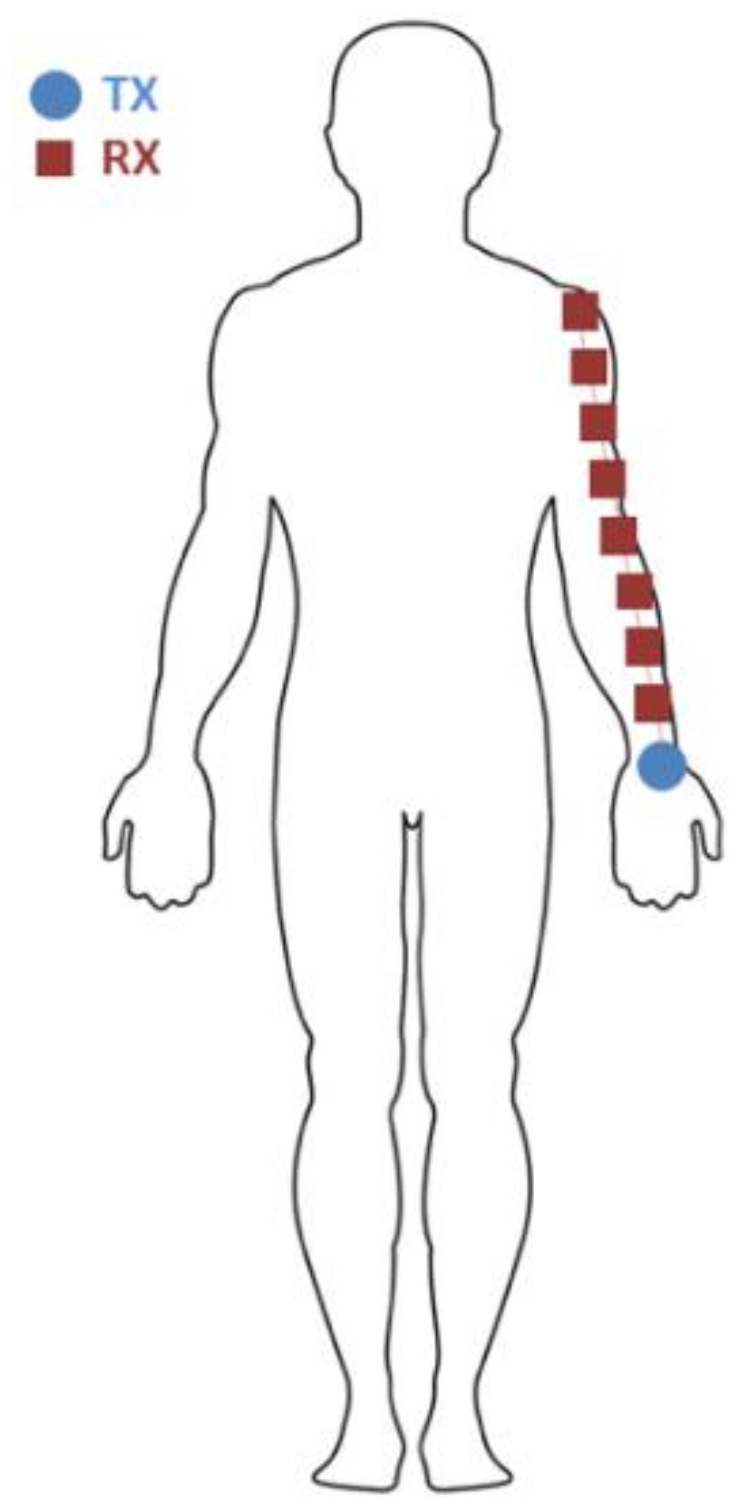
Frontal view of the on-body setup of the reference path-loss measurement scenario. The blue marker indicates the TX location, while the red markers indicate different RX positions.

The goal of the reference measurement is to compare narrowband path loss as a function of distance in a fixed scenario using various wireless technologies for on-body communication at different frequencies. The IEEE 802.15.6 standard for on-body communication considers frequencies from 400 MHz–3.5 GHz. In this frequency span, we have performed measurements from 420 MHz–3 GHz, with different wireless technologies per studied frequency. Path loss (or equivalently path gain) is a commonly studied quantity in (on-body) RF propagation and is the ratio of the received power and the input power, usually as a function of distance or frequency [34]. Note that in the definition used in this paper, path loss expressed in decibels is negative. Generally, a power loss should be subtracted in a power balance and consequently path loss should be positive in decibels. However, to keep formatting in line with literature and S-parameter measurements, path loss is reported in this paper as negative values in decibels. It should be noted that other authors also refer to the same quantity as path gain. Additionally, it should be noted that the International Telecommunication Union (ITU) recommends the use of the term system loss instead of path loss [35], referring to the loss between antenna or electrode terminals instead of the loss purely caused by propagation.All the studied wireless, on-body technologies are studied in the same reference scenario shown in Figure 1. A transmitting node (TX) is placed on the left wrist of a male subject (30 y old, height 191 cm, mass 83 kg) in upright anatomical position. Consequently, the TX position was 93 ± 5 cm above the floor. A receiving node (RX) is placed on the left arm at different separation distances (*d*) from the TX. This path loss is measured over distance, where distance was determined using tape measure from aperture to aperture along the arm and is varied from 10 cm to 50 cm in steps of 5 cm. All measurements are executed in an indoor environment. The parameters and (additional) configurations used for every studied technology are listed in the next sections. This reference scenario is chosen because it represents a commonly targeted on-body application where a wearable device or a prosthetic is worn on the wrist and needs to communicate along the arm with another body-worn RF node. As a note, three-dimensional radiation characteristics of the used technologies are not reported, given the variable influence of the body on these parameters.

Each of the wireless technologies has a different method of (indirectly) registering received powers on the RX nodes. Eventually, these are all converted to values in relative powers, i.e. ratios of the received power over the input power (*P*_dB_) in decibel. These are fed into a log-linear least-square fit for each technology using the following two path loss models [36]:

(1) $$P_{\mathrm{dB}}\left(d\right)=P_{0}-10n\cdot \log_{10}\left(\frac{d}{d_{0}}\right)+N\left(0,\sigma_{p}^{2}\right),$$

with $P_{0}$ the path loss at the reference distance $d_{0}$ of 10 cm, d the separation between TX and RX, *n* the path loss exponent, and $N\left(0,\sigma_{p}^{2}\right)$ is a term quantifying the (zero-mean) lognormal variance on the path loss. $\sigma_{p}$ is the standard deviation on the path loss model. This path loss model is used frequently when quantifying (on-body) path loss measurements in indoor environments [36]. While this model is simplistic, it uses only three parameters, and is therefore easier to interpret physically and allow for an intuitive comparison over different frequencies and technologies. An alternative model is given by:

(2) $$P_{\mathrm{dB}}\left(d\right)=P_{0}+10\cdot \log_{10}\left(e^{-m_{0}d}\right)+N\left(0,\sigma_{p}^{2}\right),$$

This model uses an exponential loss in power as function of distance. $m_{0}$, is the loss per unit of distance. $N\left(0,\sigma_{p}^{2}\right)$ is again a term quantifying the lognormal variance on the path loss and $P_{0}$ is the baseline path loss.

These path loss models are fitted to our measured data per technology and frequency. Path loss is in general a good proxy for other important link parameters such as communication range, packet delivery ratio, coverage, required transmitted power, etc. [17]. Minimizing the influence of path loss is therefore an important aspect of optimizing wireless links. The studied path loss models describe the on-body channels’ large-scale characteristics [34]. The small-scale characteristics of the on-body channel are not studied in this manuscript. 

### 2.2. RF Technologies

The studied RF technologies are described in the following subsections.

#### 2.2.1. 915 MHz Printed Folded Dipoles for UHF RFID

UHF RFID is a wireless technology that operates in a frequency band around 900 MHz (in the United States: 902–928 MHz) and is mainly used for passive wireless identification of objects. However, the new generation of the UHF RFID standard also allows for passive transmission of sensor data from the tag, i.e., the remote, passive antenna-IC-sensor system, towards a reader, i.e., a powered system with an antenna and an RFID compatible radio [17]. This makes this technology an interesting option in WBANs, since it allows one to place the energy source in a central hub and work with passive tag(s) on the other side of the link.

**Figure 2:**
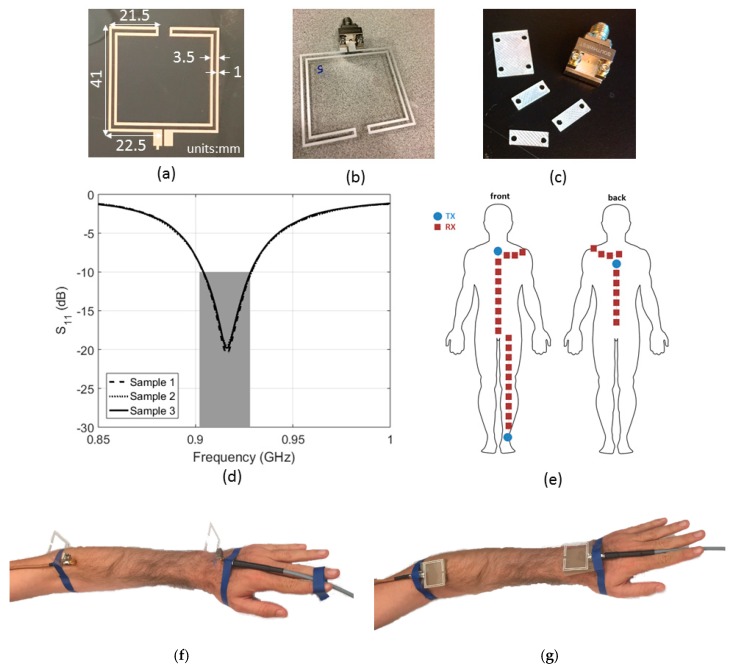
(**a**) Screen-printed folded dipole on PEN with antenna dimensions (mm). (**b**) Screen-printed folded dipole with the used mechanical SMA connector (**c**) The used mechanical SMA connector with the printed plastic spacer. (**d**) Measured S_11_ of three printed antennas (black) and the UHF RFID frequency band (grey). (**e**) Additional on-body path-loss configurations used for measurements using this antenna. (**f**) On-body setup in the reference scenario with antennas orthogonal to the skin surface and (**g**) On-body setup in the reference scenario with antennas parallel to the skin surface.

We have used screen-printed, dipole antennas with a free-space power reflection coefficient (S_11_) below −10 dB at 915 MHz according to the design presented in [37]. Dipole antennas are frequently used in UHF RFID tags [38]. The antennas are printed on a 125 μm-thick polyethylene naphtalate (PEN) substrate (Q65HA from Teijin Dupont Films, Wilmington, DE, USA) [39] using Creative Materials conductive Silver ink no. 12633 [40]. The antennas are fed using a mechanically connected SMA connector (292-64A-06, Centric RF, Allen, TX, USA) which is adapted using a 0.96 ± 0.02 mm thick plastic spacer underneath the center coax in order to eliminate the connector’s capacitance. Figure 2 shows the screen-printed antennas, the connectors with spacer, their dimensions, and their measured S_11_. The antennas were used to perform on-body path-loss measurements at 915 MHz. Two antennas were placed on the positions shown in Figure 1. A cardboard spacer with thickness 2.83 ± 0.03 mm backed the antennas. The antennas were placed on the body in two configurations: orthogonal to the skin and parallel to the skin, see Figure 2f,g. In the orthogonal case, the connector and a 90° SMA connector introduce 26.8 ± 0.1 mm spacing in between the feed point of the antenna and the cardboard spacer. In the parallel case the connector introduces an additional 4.42 ± 0.11 mm spacing from its bottom until the coax.

A VNA (Agilent N5242A, PNA-X, Santa Clara, CA, USA) was used to register two-port S-parameters (S_11_, S_12_, S_21_, and S_22_). For each position, 50 measurements were executed from 865 MHz to 965 MHz with 501 frequency steps (sweep time 5.3 ms). The S_21_ and S_12_ measurements were pooled and their average value (calculated in decibels) was determined as a function of separation distance (*d*), together with the standard deviation on this value. The 100 S_12/21_ samples were used as the input for the path loss models listed in Equations (1) and (2). In addition to the reference scenario, the antennas at 915 MHz were also used to assess on-body path loss in four additional scenarios on the body (see Figure 2e):

(1) Chest: TX above the sternum and RX moved down in steps of 5 cm
(2) Back: TX in between the shoulder blades and RX moved down in steps of 5 cm
(3) Leg: TX on the ankle and RX moved upward in steps of 5 cm
(4) Around the torso: TX on the sternum and RX moved around the shoulders on the same height in steps of 5 cm.

The goal of these measurements was to compare the reference scenario to different on-body scenarios using the same frequency and antennas. During these measurements, we used the same antennas, VNA, on-body setup, statistical processing and path loss model as described above for the reference scenario.

#### 2.2.2. 450 MHz Body-Coupled Communication and On-Body Monopoles

**Figure 3:**
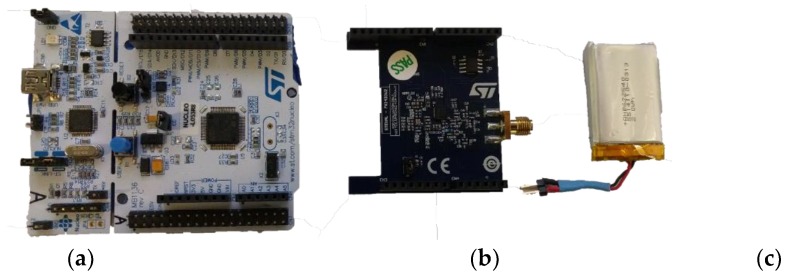
(**a**) Mother board: Nucleo STM32L053R8, (**b**) daughter board with ST S2-LP sub-GHz radio, (**c**) 3.6 V battery.

Body Coupled Communication (BCC) should only be tested with isolated power supplies such as battery-powered instruments to prevent coupling. Additionally, the size of the instrument’s ground plane should be minimized to limit over-the-air coupling via the instrument. This is because a bigger reference plane will increase the coupling of the system to its environment. Consequently, it was chosen to use the small form factor evaluation kit STEVAL-FKI433V2 [41] (STMicroelectronics, Geneva, Switzerland). This kit consists of an S2-LP radio daughterboard plugged onto a Nucleo Board STM32L053R8 (Figure 3a,b). The latter embeds a Cortex-M0+ that controls the radio and allows data download to a computer for processing. Both boards are powered by a single 3.6 V battery (Figure 3c). For our experiments, two of these modules are used: one for transmission and one for reception.

**Figure 4:**
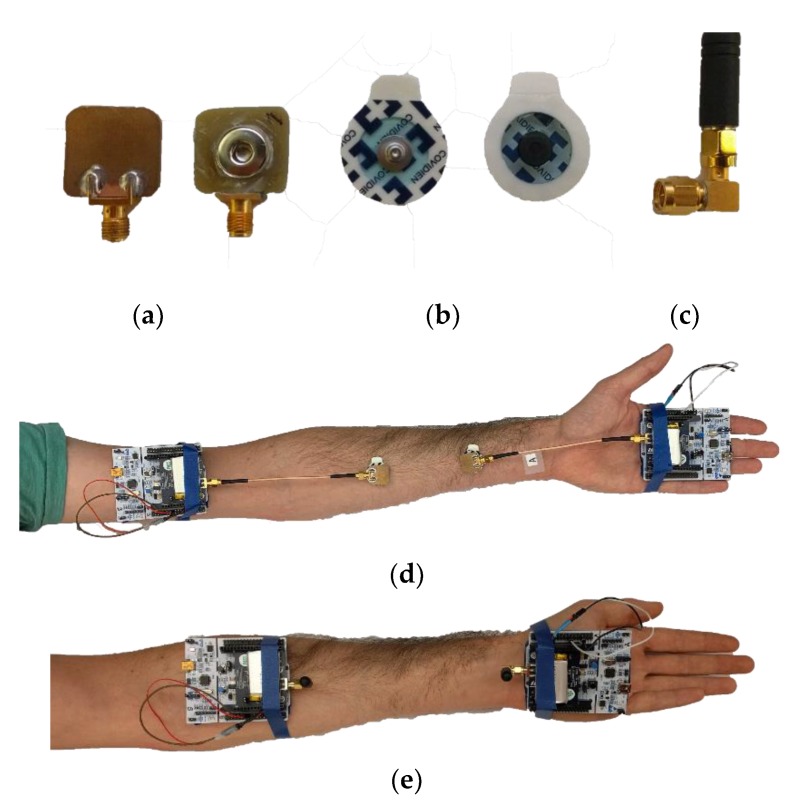
(**a**) BCC electrodes (connector part), (**b**) pre-gelled electrodes, (**c**) 450 MHz monopole antenna from the evaluation kit. (**d**) BCC on-body, (**e**) 450 MHz monopole antenna on-body.

The BCC electrodes are composed of two distinct parts. On one side is a 2 cm by 2 cm backplane that consists of a single copper layer PCB. On the insulating side, a snap is glued. Both are wired to an SMA connector, as depicted in Figure 4a. The second part is a pre-gelled standard medical electrode from Covidien (Kendall disposable electrode, Arbo H124SH [42]) which can be snapped in the BCC electrode described above (Figure 4b). A 15 cm long SMA cable directly connects the electrode to the radio, with no matching network added. For comparison purposes, a pair ofwire antennas in a monopole type package (see Figure 4c) were also used to perform the same tests. The antennas were included in the evaluation kit STEVAL-FKI433V2 with the sub-GHz radio and the Nucleo board and exhibit an S_11_ value of −8 dB at 450 MHz. The antennas were directly connected (no matching) to the same transceivers used for BCC for the sake of comparability between both measurement setups. via a 90° SMA connector (Figure 4c), positioning them perpendicular and 2 cm above the surface of the arm.

In order to measure the attenuation of the channel under different conditions, the transmitter radio (TX) was set up to feed a continuous wave (CW) at the frequency range of interest: 420 MHz to 510 MHz with steps of 10 MHz. The receiver radio (RX) was set up to store the received signal strength indicator (RSSI) values in the flash memory of the microcontroller on the motherboard. Because the TX and RX are asynchronous both boards follow a 5 s cycle for a given frequency. Once the cycle begins, the TX waits 750 ms before transmitting a continuous wave, lasting 3 s. At the end of the 5 s cycle, the TX switched to the following pre-programmed frequency and waits for the start signal of the following cycle. On the RX side, as soon as the program is triggered, the board begins saving the value of the RSSI register in the Coretex-M0+ RAM every 40 ms. A total of 116 samples are collected per frequency step. This phase lasts for 4.64 s. At the end of the cycle, the data saved in the RAM are transferred to the flash memory of the microcontroller. The radio switches to the following pre-programmed frequency and awaits the start signal.The timing of start signals is kept fixed by interruptions from the on-board timer, and the measurement is triggered manually one-time by an operator.. The flexibility of this approach and the accuracy of the timer offer a certain flexibility, relaxing the synchronization constraint on the operator triggering the initial start signal on both TX and RX at the same time. 

Prior to the measurement, a relative power calibration was performed. It consists of setting the output power of the TX radio to 0 dBm (an inaccurate value when measured with a precision instrument) and recording the RSSI on the RX radio for all the frequencies of interest. The radio offers a function that converts the RSSI to dBm. In the end, the measured received power is 3 to 6 dB lower that the TX settings (the 0 dBm output power). Those losses are mainly due to the on-board matching network centered around 433 MHz.

All tests are performed three times under the same conditions for repeatability purposes and then average for plotting. We explored multiple distances between electrodes on a human arm for 10 different frequencies. In order to compare the impact of the human body on the communication channel, free-space measurements (with the same BCC electrodes) were also made. The same subject was standing with both arms along the body (vertically). No other pre-gelled electrode but the two required ones were on the body for each measurement (see Figure 4d,e). The tests with the antennas provided by the manufacturer were performed under the same conditions.

#### 2.2.3. Bluetooth Nodes at 2.4 GHz

Bluetooth is a wireless technology that operates between 2400–2485 MHz. The technology is widely used by for short-range communication among personal wireless user devices and for wearable sensor applications. A low-power consumption standard, Bluetooth low-energy (BLE), exists and is used often as a standard competing with IEEE 802.15.6 for wearable devices.

**Figure 5:**
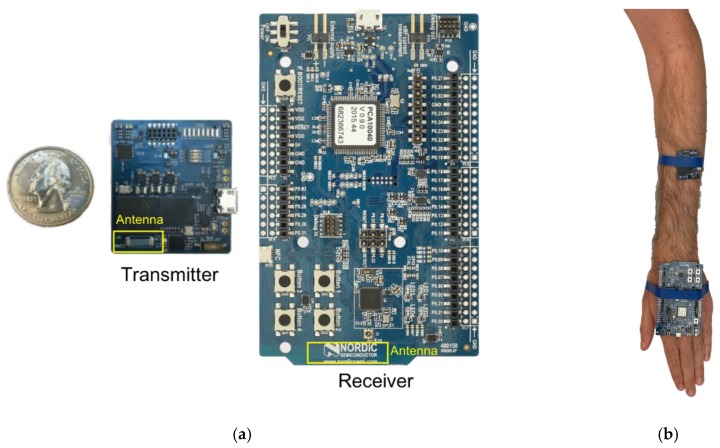
(**a**) Bluetooth nodes with the transmitter node shown on the left and the receiver on the right. (**b**) Bluetooth nodes measurement setup.

Two Bluetooth nodes, one custom-made transmitter [43] and a commercial receiver, were placed on the on-body locations shown in Figure 1. The RX node registered RSSI values in dBm over time, which are treated as a proxy for received power in this study. 100 samples are retained per TX-RX separation distance and fed into Equations (1) and (2) in order to fit the path loss models. Table 1 shows the transmitter and receiver specifications for these measurements. Figure 5 shows the used nodes with indicated antenna locations. Fully operational Bluetooth nodes were used instead of antennas at this frequency in order to study a complete wireless system.

The boards were placed on the subject’s arm in the measurement configuration illustrated in Figure 5b. The boards were placed parallel to the skin, with the antennas facing each other. Once worn on the body, the RX antenna was at a distance of 3.9 mm from the skin, while the transmitter was at a distance of 6.9 mm from the skin.

#### 2.2.4. Monopoles at 2 GHz

A major cause of antenna underperformance in WBANs is antenna detuning due to proximity to the human body. Shielded monopole antennas could overcome this issue if the ground plane is aligned with the body. For this research, path loss measurements are performed with radially symmetrical shielded monopoles, tuned at 2 GHz. This frequency was selected because it results in a considerably smaller antenna size in comparison to 420 MHz or 900 MHz antennas. The 2.4 GHz ISM band was avoided to prevent interference during our measurements. Furthermore, the 2 GHz monopoles are compatible with the antenna arrays used in this research, for which the frequency selection was driven by component specifications.

**Figure 6:**
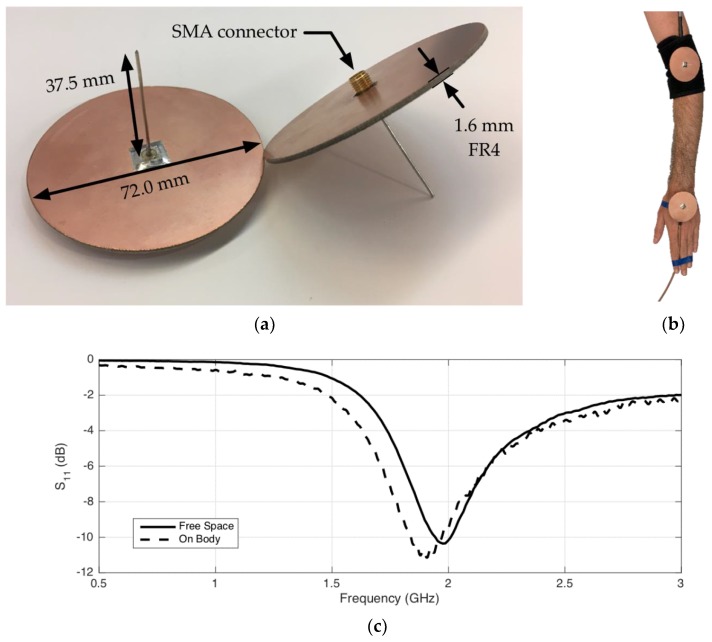
(**a**) Radially symmetrical shielded monopole with antenna dimensions (mm). (**b**) Reference scenario with two on-body monopoles, connected with 90º SMA connectors. (**c**) Measured Power reflection coefficient (S_11_) of the monopole in free space (solid black) and on the body (dashed black).

The design of the antenna is presented in Figure 6a: A 37.5 mm monopole sticks out of a 72.0 mm FR4 disk with a double-sided copper ground plane. The antenna is equipped with an SMA connector. For on-body applications, a 90° SMA connector is used, allowing close-to-skin mounting, as depicted in Figure 6b.

Three identical shielded monopoles were manufactured, allowing 2-port measurements of the reference scenario, and 3-port measurements of configurations with on-body repeaters. For all S-parameter measurements, the VNA described above is used with 50 ohm ports. Each configuration is measured 50 times with a frequency span of 500 MHz–3 GHz with 501 frequency steps (sweep time 5.3 ms). The S-parameters are then used as an input in the path-loss models. Figure 6c presents the monopole’s S_11_ parameters in free space, and mounted on the body. In the free space scenario, resonance can be observed at 2 GHz, and the intended −10 dB S_11_ value is obtained. When the antenna is mounted on the body, the resonance peak shifts towards 1.91 GHz. However, due to the wide resonance peak, the S11 value at 2 GHz is still −9.5 dB.

**Figure 7:**
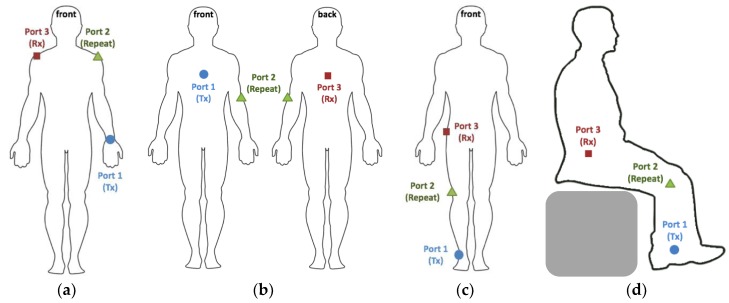
On-body repeater test scenarios: (**a**) wrist to opposite shoulder, (**b**) chest to back, (**c**) ankle to hip in upright pose, and (**d**) ankle to hip in sitting position.

In addition to the reference scenario, the monopole antennas were also used to evaluate the performance of on-body repeaters in 4 additional scenarios on the body, as depicted in Figure 7.

(a) Wrist to opposite shoulder with repeater on the shoulder in upright anatomical position
(b) Chest to back with a repeater on the left elbow in upright anatomical position.
(c) Ankle to hip communication with a repeater on the knee in upright position.
(d) Ankle to hip communication with a repeater on the knee in sitting position (obstructed Line-of-Sight (LOS) between RX and TX).

To this aim, we executed three port measurements with three monopoles placed simultaneously on the body. All nine S-parameters were registered 50 times in each configuration using the VNA settings described above.

#### 2.2.5. Dipole Arrays at 2 GHz

**Figure 8:**
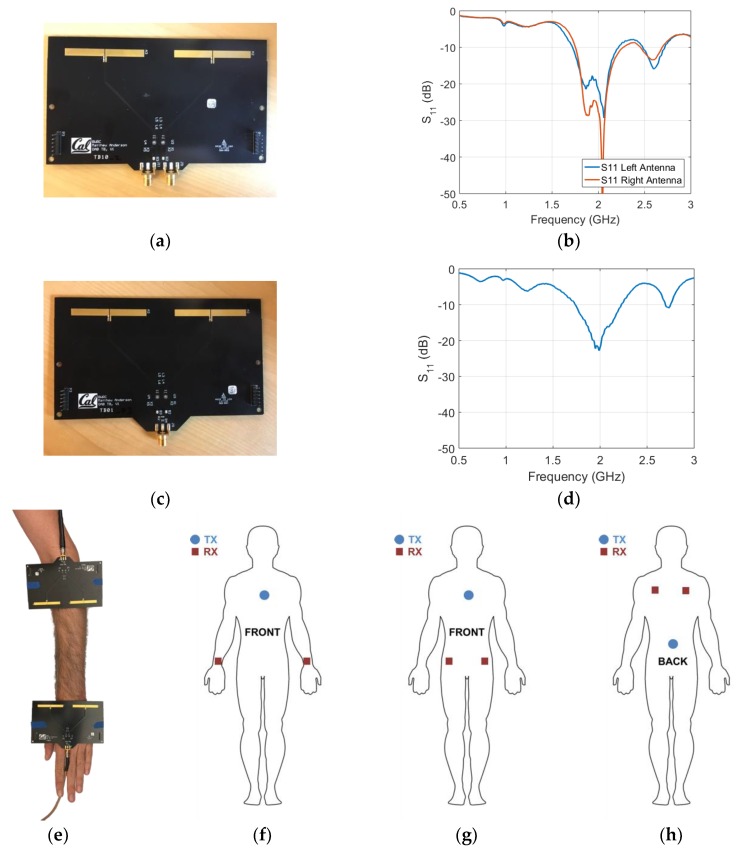
(**a**) Phased two-port antenna array used for measurements at 2.05 GHz. (**b**) Power reflection coefficient of both dipole antennas in the array. (**c**) Power-combined antenna array used for measurements at 2.05 GHz. (**d**) Power reflection coefficient of the power-combined antenna array. (**e**) Array placement on the body. The shown boards are the power-combined arrays. (**f**) On-body beam steering scenario 1 (**g**) On-body beam steering scenario 2 (**h**) On-body beam steering scenario 3.

To evaluate the on-body performance of RF antenna arrays, a linear antenna array consisting of two 2.05 GHz half-wavelength dipoles was used. The array has an antenna-to-antenna spacing of 75 mm (or approximately half a wavelength) and is arranged to maximize broadside radiation and minimize antenna interaction. Details of the array design can be seen in Figure 8a. The dipoles are designed as rectangular copper traces on a standard FR4 material (Isola FR370HR, Chandler, AZ, USA) with a relative dielectric constant of roughly 4.1 at 2 GHz and thickness of 1.6 mm. This results in dipoles of length 54.0 mm, width 5.0 mm and feed gap of 0.5 mm. A series capacitance of 3.5 pF is used to match the antennas’ differential impedance to 50 Ω and each matched dipole antenna is fed via a approximately one-wavelength long differential micro strip transmission line and terminated with an edge mount SMA connector. Each antenna in the phased array can be driven independently via its own SMA connector to steer the array’s beam. These arrays were leveraged from the work done in [44]. Dipole antennas are chosen because they are an efficient solution for planar, uniform, linear arrays. 

An adapted board was also developed with the same antennas, antenna spacing and transmission line layout as the phased array but with the addition of a SMT power splitter/combiner (Figure 8c). With the power splitter, the phase relationship between both antennas on the second array is fixed at zero and a single SMA connector can be used to feed the array. This power-combined array was used for the reference path loss measurement scenario.

The antennas free-space S_11_ is significantly lower than −10 dB at the desired RF frequency, 2.05 GHz for both array types. This provides strong validation for the array design. The measured S-parameters for each of the antennas in the phased array is shown in Figure 8b,d.

Two (power-combined) boards were placed on the body in the reference scenario with the antennas facing each other, as seen in Figure 8e. The S-parameters were registered using the same VNA settings as described in the previous subsection. These S-parameters were then used as an input in the path-loss models. In addition to the reference scenario, the dipole arrays were also used to evaluate the performance of on-body beam steering in three additional scenarios on the body:

(1) Phased Array placed on the chest (sternum) to both hips with the power-combined arrays.
(2) Phased Array placed on the chest (sternum) to both wrists with the power-combined arrays.
(3) Phased Array placed on the lower back (centered on spine) to shoulder blades with the power-combined arrays.

The phased array board was placed on the body in the configurations described above and connected to two ports of the VNA (ports 1 and 2). Simultaneously, two power-combined arrays were placed on two other locations on the body and connected to the VNA (ports 3 and 4). 4 port measurements registering all 16 S-parameters were executed 50 times in each configuration using the VNA settings described above. The relative phase difference (Δϕ) between the two ports on the board were adapted from 0°–360° in order to see the variation of received power on the two power combined boards ($P_{r,3}$ and $P_{r,4}$):

(3) $$P_{r,j}=\frac{P_{\mathrm{out}}}{2}\cdot \left[S_{1j}+S_{2j}e^{i\Delta \phi }\right]\times {\left[S_{1j}+S_{2j}e^{i\Delta \phi }\right]}^{*}$$

with *P*_out_ the VNA’s output power and j∈[3,4].

## 3. Results

This section presents the measurement results. An overall comparison of path loss measurements and models for different RF technologies in the reference scenario is presented. This comparison is followed by sections discussing the results obtained for the specific RF technologies and scenarios. 

### 3.1. Path loss in Reference Scenario (Left-Arm)

**Figure 9:**
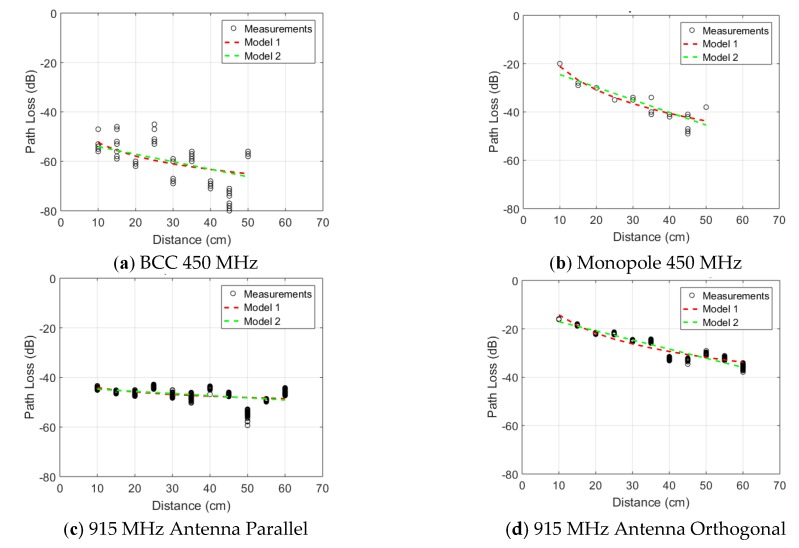

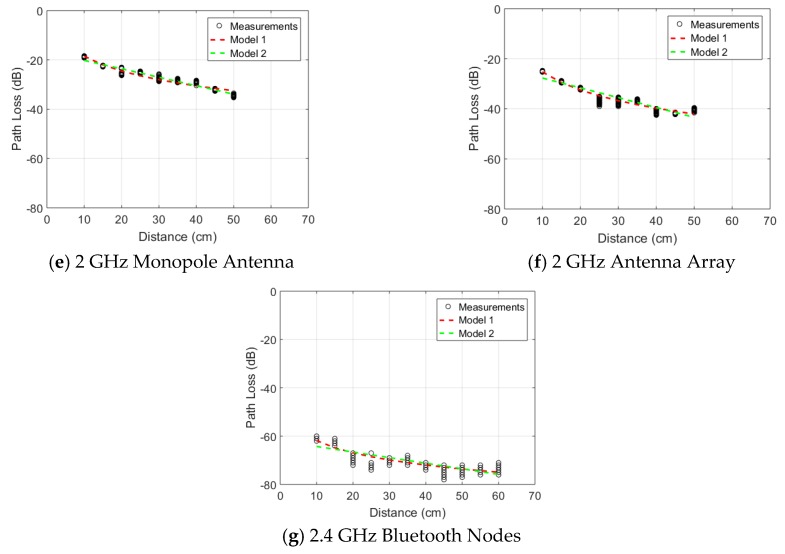
Measured path loss as a function of distance in the reference scenario (left arm). Measurements are shown as black markers, alongside two fits using the two considered path loss models for the seven studied technologies: (**a**) 450 MHz monopole, (**b**) 450 MHz BCC, (**c**) 915 MHz folded dipole parallel to the body, (**d**) 915 MHz Folded dipole orthogonal to the body, (**e**) 2 GHz Monopole antennas, (**f**) 2 GHz Dipole Arrays, and (**g**) 2.4 GHz Bluetooth nodes.

Figure 9 shows the measured path loss in the reference scenario shown in Figure 1 for all seven studied technologies. Following model 1, $P_{0}$ ranges from −14 dB for the folded dipole antenna at 915 MHz down to −62 dB for the 2.4 GHz Bluetooth nodes. Based on model 2, $P_{0}$ varies in a similar range from −13 dB down to −62 dB for the same technologies. The path loss exponent, *n* (only in path loss model 1) varies from 1.7 for Bluetooth up to 3.2 for the 450 MHz monopole. A lower value than other technologies was obtained with the antenna parallel to the body at 915 MHz where *n* is 0.57. The attenuation per unit of distance, $m_{0}$, in model 2, ranges from 0.23 dB/cm up to 0.52 dB/cm for the Bluetooth nodes and the 450 MHz monopole, respectively. Similarly to the path loss exponent, the 915 MHz parallel antenna technology shows a significantly lower value of 0.087 dB/cm. The lognormal standard deviation according to model 1 ranges from 1.3 dB at 2 GHz to 6.2 dB for BCC operating at 450 MHz. In model 2, these standard deviations go from 1.1 dB for the 2 GHz monopole to 6.2 dB for BCC as well.

**Figure 10:**
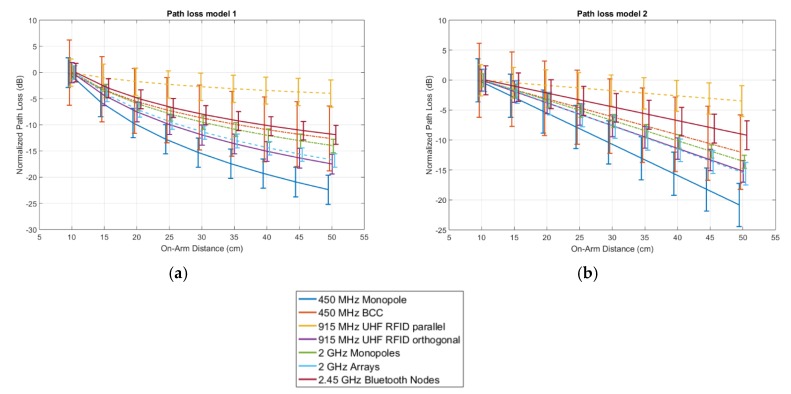
Normalized path loss models as a function of distance in the reference scenario (left arm) for the seven studied configurations: (**a**) Model according to Equation (1), (**b**) Model according to Equation (2). The solid lines indicate the models described in Table 2, while the whiskers are standard deviations (whiskers are shifted slightly to increase figure clarity).

Figure 10 shows the path loss, normalized to *P*_0_, for the seven studied conditions in the reference scenario.

Excluding the measurements at 915 MHz with the antenna parallel to the skin, all path loss curves are clustered along a similar trend. None of the path loss models exceed 35 dB over 40 cm propagation along the arm.

### 3.2. On-Body Measurements at 450 MHz using BCC and Monopole antennas

**Figure 11:**
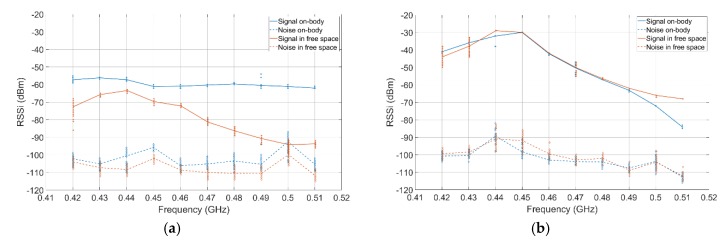
Attenuation over frequency with electrodes (**a**) and monopole (**b**) in free space (air) versus on-body for a 20 cm range. Dashed lines represent the noise floor. The solid curves show the average values at each frequency while the markers show the individual measured samples.

In Figure 11a the electrodes (BCC) were positioned 20 cm apart, in free space and on-body. The on-body results were improved by at least 20 dB compared to free space. The on body noise was slightly degraded by a maximum of 10 dB. Figure 11b compares the antenna results in free space and on-body. The results are very similar except for the highest part of the spectrum where the on-body attenuation is greater.

In the antenna configuration, for a 20 cm communication, the path loss of the channel is very similar to free space. This is due to the size of the antenna and its distance with the human body. However, with electrodes for BCC, the body improves the communication channel compared to free space as well as offering a flatter response in frequency.

### 3.3. On-Body Measurements using 915 MHz UHF RFID Antennas in Five Scenarios

**Figure 12:**
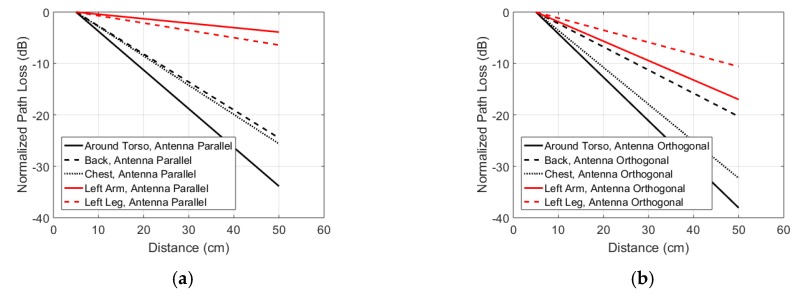
Measured normalized path-loss according to the path loss model described in Equation 2 at 915 MHz in five different on-body scenarios for (**a**) folded dipole antennas parallel to the body and (**b**) folded dipole antennas orthogonal to the body.

Figure 9c,d show the path loss measurements in the reference scenario illustrated in Figure 1 for the printed antenna parallel and orthogonal to the skin surface, respectively. The baseline path loss at 10 cm (minimal separation distance) is significantly higher in the case of parallel antennas, while the path loss exponent (model 1) or path loss per distance (model 2) are significantly higher for the orthogonal antennas. Figure 12 shows the fitted path loss model 2, normalized to P_0_, for the five studied on-body propagation scenarios at 915 MHz. Table 3 lists the parameters of the path loss model. Propagation along the arm (parallel configuration) and the leg (orthogonal configuration) experienced the least path loss of the five studied scenarios. Around the torso propagation results in the highest path loss exponent.

### 3.4. Monopoles as On-Body Repeaters at 2 GHz

**Figure 13:**
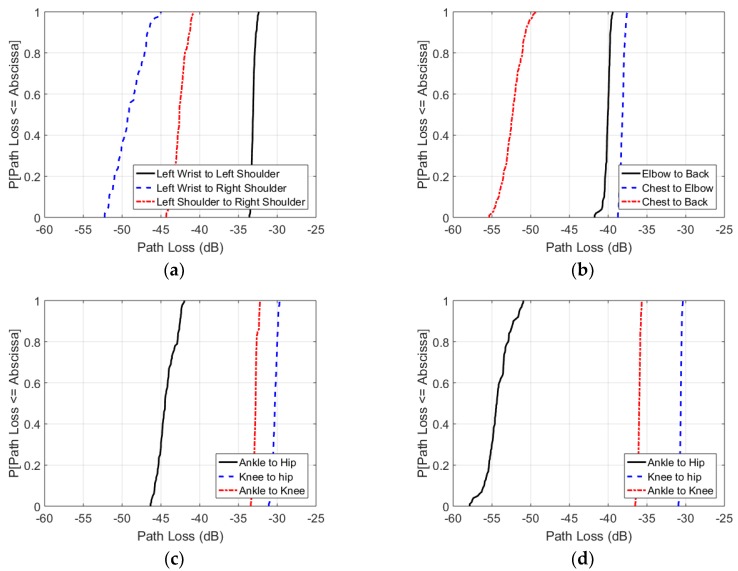
Cumulative distribution function of on-body path loss in between monopole antennas at 2 GHz in four on-body scenarios: (**a**) Standing upright with monopole antennas on the left wrist, left shoulder, and right shoulder; (**b**) Standing upright with monopole antennas on the chest (sternum), left elbow, and back (in between the shoulder blades); (**c**) Standing upright with monopole antennas on the ankle, knee, and hip; (**d**) Sitting with monopole antennas on the ankle, knee, and hip.

Figure 13 shows the cumulative distribution functions of the path loss measurements for all repeater scenarios, as illustrated in Figure 7. Figure 13a shows that a path from left wrist to left shoulder and left shoulder to right shoulder results in a path loss of respectively −32 dB to −34 dB and −41 dB to −44 dB, compared to −45 dB to −53 dB for the direct path. The wrist to shoulder values match the independent measurement results of the reference scenario in Figure 9e. Figure 13b presents path loss from chest to elbow and elbow to back of respectively −37 dB to −38 dB and −39 dB to −42 dB. The direct connection from chest to back presents a path loss of −49 dB to −56 dB. Figure 13c depicts an ankle to knee and knee to hip connection of respectively −3 dB to −34 dB and −30 dB to −32 dB in upright position. The connection without the repeater on the knee has a path loss of −42 dB to −47 dB. The results for the same antenna setup with the subject in sitting position are presented in Figure 13d, illustrating an ankle to knee path loss of −35 dB to −37 dB and a knee to hip path loss of −30 dB to −32 dB. The obstructed connection from ankle to hip results in a −52 dB to −58 dB path loss.

Assessing the results of all experiments, one can remark that the paths from the TX and RX towards the repeater are always less lossy than the path between TX and RX. Furthermore, the connections to a repeater exhibit spreads of maximum 3 dB, while the TX-RX connections show spreads of 5 dB to 8 dB.

Figure 13 also demonstrates that the connection from wrist to shoulder is less lossy than the connection from ankle to hip. Furthermore, in upright positions the highest path loss is observed for the chest-back scenario, which matches the 915 MHz measurement results.

### 3.5. On-Body Beam Steering at 2 GHz using Antenna Arrays

**Figure 14:**
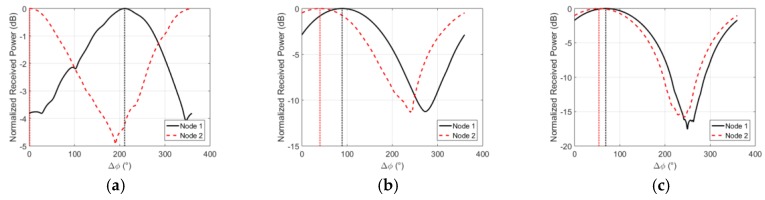
Normalized received power on the power-combined on-body arrays in three studied scenarios shown in Figure 8g–h. (**a**) On-body beam steering scenario 1 (**b**) On-body beam steering scenario 2 (b) On-body beam steering scenario 3.

Figure 8f to h shows the three on-body scenarios used for evaluating the effect of beam steering. The phased two-port antenna array was used for the TX node and the power-combined arrays were used for nodes 1 and 2. In all scenarios, the TX node is between 28 and 35 cm away from nodes 1 and 2. As the relative phase between the left and right antenna (Δϕ) is adjusted from 0° to 360°, the TX node’s beam angle is swept through all supported angles. The received power at nodes 1 and 2 for all Δ∅ is shown in Figure 14.

### 3.6. On-Body Measurements using 2.4 GHz Bluetooth Nodes

Figure 9g illustrates the measured path loss for the reference scenario versus distance. The baseline value (for 5 cm distance) shows the highest path loss among all technologies. This is primarily due to using ceramic and PCB antennas, which are optimized to achieve minimal board form factor at the cost of higher loss. Moreover, the distance between the antenna and skin is the smallest among all setups. However, since we are interested in the normalized path loss comparison (Section 3.1.6), the only required condition is that the lowest received signal be above the receiver sensitivity. An improved path loss can be achieved in this technology by utilizing more optimized antennas, i.e., optimizing *P*_0_.

## 4. Discussion

### 4.1. Comparison of Path Loss over Distance for Different Technologies

Two different on-body antenna configurations are studied at 915 MHz: antennas parallel or orthogonal to the body surface. The orthogonal configuration leads to a relatively high transferred power at 5 cm, when compared to the other studied technologies, but experiences more loss over distance and a higher path loss exponent than the parallel configuration, which showed the lowest loss over distance for all studied configurations. We attribute the high $P_{0}$ (low loss) in the orthogonal case to the increase separation distance of the antenna to the body [27] and a reduction in current cancellation in comparison to the parallel configuration [45]. We attribute the lower path loss exponent and loss per distance in the parallel configuration to the higher baseline loss experienced in this scenario, which increases the relative importance of specular and diffuse components coming from indoor reflections [26], whose magnitude is independent of separation distance. The parallel folded dipole at 915 MHz shows a different behavior from all the other technologies analyzed in this study. It is assumed that the communication is mostly multipath. This is justified by the combination of one of the lowest baseline path loss and the lowest attenuation over distance, which is three times lower than the second best result.

The same antennas were also used to measure path loss along four other channels along the body of the same subject. These measurements show that the reference scenario, along the arm, at 915 MHz is one of the two scenarios with the lowest path loss per unit of distance or the lowest path loss exponent. An increase in the path loss over distance and path loss exponent with an increased proportion dielectric material in the studied channel is obeserved. Around body propagation results in the highest path loss exponent and loss per distance. This is in line with literature [28,32] where around body propagation is generally found to have the higher path loss exponents or the highest loss per distance.

The baseline path loss ($P_{0}$) varies severly between the different studied technologies. In general, those configurations with an antenna orthogonal to the skin surface (monopole at 450 MHz, printed antenna orthogonal to skin at 915 MHz, and monopole at 2 GHz) have a lower initial path loss than the other studied techniques at the same frequencies. $P_{0}$ is similar for the the 2 GHz antenna arrays and the 2 GHz monopole. Their size and operating frequency being similar, these results were expected. In addition, their attenuation per unit distance is also in the same range. The same observation is made for Bluetooth. However it suffers of a higher initial loss as explained in Section 3.1.5. 

The sub-GHz RF orthogonal technologies (915 MHz antenna orthogonal and 450 MHz monopoles) provide the highest $P_{0}$. This is explained by three factors: lower frequency of operation, the optimized configuration where both antennas’ linear polarizations were parallel and a relatively high distance between antenna and body due to the test setup decreasing the impact of the body on the measurement, as shown in [27]. Figure 9 and Table 2 highlight two groups: technologies well described and fitted with our theoretical model (Figure 9b,g) and the technology, BCC (Figure 9a), where the results spread is larger. This effect can be observed at discontinuities (elbow and shoulder) in the medium since BCC uses the body itself as a communication channel. BCC, due to its topology with no matching network due to the direct on-body contact and communication mechanism, presents one of the lowest $P_{0}$. BCC appears less efficient than regular RF in a line of sight configuration. Its attenuation over distance is greater but its linearity over a wide frequency band is higher. In addition, the electrodes used offer a smaller form factor, are parallel to the body and do not require any kind of matching network. 

An important observation is that none of the studied technologies show an average path loss of more than 35 dB over a propagation distance of 40 cm, see Figure 11. Simultaneously, the differences in $P_{0}$, even within the same frequency, can be larger than this added path loss over 40 cm. This indicates that selection of a proper antenna/electrode configuration is extremely important in designing an on-body link.

### 4.2. Comparison with Literature

When comparing the measured path loss exponents (see Table 2) with the IEEE channel model [21,46], our measurements at 915 MHz (*n* = 1.8–2.6), 2 GHz and 2.4 GHz (*n* = 1.7 to 2.4) fall in between the indoor and anechoic measurements presented in the channel model. The IEEE channel model lists values for *n* of 1.6 (indoor) and 2.9 (anechoic) at 915 MHz and 0.66 (indoor) and 2.9 (anechoic) at 2.45 GHz. The path loss exponents measured in this study at 2–2.4 GHz, fall in between the ones found in literature at the same frequency span: *n* = 3.35 [26], *n* = 4.7 [47], *n* = 2.5–4 [27], *n* = 2 [22], and *n* = 0.86 [48]. In terms of loss per distance *m*_0_, our measurements at 915 MHz show a lower value in the reference scenario (0.1–0.3 dB/cm) than what is shown in literature: 0.4 dB/cm [32,36] and 0.74 dB/cm [47]. We attribute this lower loss per distance in our model to the fact that [32,36,47] also consider other locations on the body than the arm. Our measurements (see Figure 12) show that the path loss per distance along the arm is lower than on other parts of the body at 915 MHz. The loss per distance at 2–2.4 GHz (0.23-0.39 dB/cm) compares excellent to literature: 0.3 dB/cm [28], 0.53 dB/cm [47], and 0.5 dB/cm [32,36]. A similar correspondence is found at 450 MHz (0.52 dB/cm for RF and 0.3 dB/cm for BCC) versus 0.3 dB/cm [28] and 0.44 dB/cm [47]. Studies have found [23] a path loss between −50 and −40 dB using monopole antennas with a ground plane in a static around the body link at 900 MHz, while we found −37 ± 5.7 dB (parallel case) and 30 ± 7 dB (orthogonal case) as path loss around the full body.

We measured lower standard deviations (see Table 2) than presented in the IEEE channel model [21,46]: 4.6 to 5.6 dB, 5.4 to 11 dB, and 3.8 to 6.9 dB at 400, 900, and 2450 MHz, respectively. However, our reference scenario contains less spatial variation in on-body positions than the channel model that includes a large set of on-body locations. Hence, one would expect a smaller standard deviation relative to the path loss model.

Our RF measurements at 450 MHz show comparable losses to what is shown in literature: We measured an *n* = 3.2 versus *n* = 3.4 in [47], *n* = 2 [48], and *n* = 0.3 to 2.3 [21]. It must be noted that the aforementioned references do not consider exactly the same reference scenario, which makes comparison difficult. The BCC measurements (*n* = 1.8) fall in between the different values listed in literature [21,47,48]. The standard deviations on our measurements are lower than what is found in literature both for BCC and RF: 4.6–5.6 dB [21,47] and 7 dB [48]. We attribute this lower variation again to the smaller variation in type of on-body locations, compared to the considered references.

Reusens et al. [26] measured P_0_ values between −32 and −41 dB at 2.45 GHz for 10 cm baseline separation. Roelens et al. [27] found P_0_ values for Equation (1) at 2.45 GHz at 10 cm separation distance between −30 and −54 dB dependent on the separation between the body and the antenna (higher separation leading to more baseline path loss). Fort et al. [32] found P_0_ values at 10 cm between −20 and −30 dB at 915 MHz and −45 to −40 dB at 2.45 GHz. In this paper, we measured P_0_ values between −12 to −44 dB at 915 MHz and −17 to −62 dB at 2.45 GHz. Note that P_0_ is also heavily influenced by the position on the body, as our measurements at 915 MHz in different on-body scenarios showed. Note that while these large variations in initial path loss observed in our study and in literature present a challenge for designing efficient WBANs, they also provide opportunities. We showed that for the same on-body configuration (on the arm) a different antenna or electode design and or configuration might provide a much better initial path loss, potentially not at the cost of an increased loss over distance. 

In [49], the attenuation for BCC at multiple distances (15 cm, 30 cm and 140 cm) is investigated in a frequency range from 1 MHz to 100 MHz. The pathloss varies from −65 dB at 1 MHz to −10 dB around 30 MHz. For these measurements, only a VNA was used in combination with baluns. Even with baluns to attempt split grounds, the VNA does not offer a fully distinct setup for TX and RX, explaining the low attenuation compared to the results provided in this work. In [50], the authors report path losses of −40 dB, −45 dB and −0 dB for BCC distances of 10, 40 and 120 cm, respectively. These results were obtained at 100 MHz with a hybrid configuration: the TX was battery powered while the RX is a spectrum analyzer. The difference between [49] and [50] could mainly be explained by a distinct ground between TX and RX in the second case. This approach for BCC is also highlighted in [51] where the authors compare grounded equipment to battery powered systems. Grounded equipment offer about 20 dB less loss than battery powered topology at 100 MHz with around −50 dB and −70 dB attenuation, respectively. These results justify our choice to measure BCC with fully battery-powered transceivers and not with a VNA.

### 4.3. Discussion on Beam steering and Repeaters at 2 GHz

#### 4.3.1. Beam Steering

Beam steering has been used on the body primarily for imaging in Magnetic Resonance Imaging and ultrasound [52,53] but, at higher frequencies (e.g. > 5 GHz) phased arrays become smaller and more viable for on-body wireless communication. The challenge with higher frequencies is the increased path loss resulting from the reduced per antenna apperature that necessitates the use of directive antenna arrays for higher gain. However, this presents an opportunity to use these antenna arrays for beam steering on the body. Beam steering can take advantage of spacial diversity to selectively communicate between different nodes on the body.

As seen in Figure 14, by sweeping the beam angle, the relative received signal power at node 1 versus node 2 can be attenuated. In the back to hip on-body senario a delta of 12 dB can be achieved between the power received at node 1 and 2. This ability to selectively amplify and attenuate the received power at spatially seperated nodes on the body can be used to simplify multiple access schemes or increase network capacity for on-body networks. Because of the variable and dynamic nature of the channel around the body, nodes that appear spatially seperated may not always show significant differences in their received power for different beam angles. This effect can be seen in the chest to wrist and chest to hip senarios where the received signal power at nodes 1 and 2 appear highly correlated over Δϕ.

Beam steering with on-body antenna arrays enables functionality in WBANs that cannot be achieved with single antennas. However, steering will consume power, which is supposed to be saved by decreasing link loss, and will require additional software or hardware. Increasing antenna gain in one direction might decrease gain for other links, this is advantageous if link selection is the goal, but can be a disadvantage if multiple links are targeted simultaneously. Finally, arrays will occupy more surface than single antennas at the same frequency.

#### 4.3.2. Repeaters

WBAN applications usually require highly reliable communications and long battery lifetimes. While star topologies have traditionally been used for WBANs, the challenging channel characteristics have spurred the investigation of multihop systems. Previous research has indicated that multihop systems can increase the energy efficiency and reduce packet error rates [54,55], especially in non-LOS (NLOS) conditions [56]. The majority of WBAN multihop papers focuses on implementations, routing protocols and energy management, whereas this research focuses specifically on the path loss in several repeater scenarios. 

The four repeater experiments presented in Section 3.1.3 support the conclusions of previous research, as the paths towards a repeater are always less lossy than the direct path from TX to RX. All repeater connections exhibit less than −44 dB path loss, while the direct TX-RX connections can be attenuated up to −58 dB. The benefit of repeaters is especially described for larger distances between TX and RX [56]. A confirmation thereof can be found in our results, considering the larger path loss along the leg in upright position, compared to the path loss along the arm. It is also demonstrated that NLOS connections between TX and RX could particularly benefit from a repeater: in the ankle to hip scenario in sitting position, a repeaterless link could have a −58 dB path loss, while the weakest signal in a link with repeater exhibits a path loss of only −37 dB. 

The experiments also indicated that the path loss values for a connection to a repeater exhibit less spread (maximum 3 dB), compared to the direct connections from TX to RX (spreads of 5 dB to 8 dB). A reduced path loss spread improves the predictability and reliability of the channel, possibly benefiting energy efficiency and reducing packet error rates.

Our measurements show that repeaters have advantages in a WBAN context. However, they also have limitations. Repeaters require additional hardware and their use might result in higher total link losses in comparison to a direct link. Additionally, working with repeaters might add interference, increase latency, and require a more complex media access control.

## 5. Conclusions 

RF path loss over distance has been measured (between 420 MHz–3 GHz) for six different technologies in a reference on-body scenario: from wrist to shoulder on the left arm. The additional path loss caused by propagating over the arm is smaller than 35 dB for all studied technologies. The baseline path loss at a minimal separation distance of 10 cm varies between −13 dB and −62 dB and can thus be tuned or optimized by selecting the right RF technology and on-body configuration. The path loss exponents (*n* = 1.7–3.2) and the propagation loss over distance (*m*_0_ = 0.23–0.52 dB/cm) are in line with literature. These values are relatively close to one another, which indicates that for propagation along the arm (10 to 50 cm) the expected path loss can essentially be considered as frequency independent in this frequency range. We have studied four other on-body scenarios at 915 MHz and found that propagation on the limbs (arms and legs) experiences significantly less path loss than propagation along the front and back of the torso. Propagation around the torso at 915 MHz results in the higher path loss. Large path losses might lead to deafness and loss of throughput in a wireless body area network. Therefore, we investigated two strategies at 2 GHz that might be employed to (dynamically) mitigate on-body path loss: repeaters and beam steering. We found that adding on-body repeaters results in reduced path loss and less spread of path loss values, especially in NLOS scenarios. Simultaneously, we found that on-body beam steering can results in 12 dB of change in the power received at spatially separated nodes for some scenarios. In future studies we will test the considered RF technologies in more on-body scenarios. The individual technologies will be further developed and implemented with application specific ICs or evaluation platforms. Finally, the ultimate goal would be the development of one solution that could handle different physical mechanisms and has the ability to switch between them.

## Figures and Tables

**Table 1:** Technical Specifications of the used Bluetooth nodes.

	Transmitter	Receiver
**Board**	Custom-Made	Nordic nRF51 Developent Kit
**Radio Chip**	nRF51822 (Nordic Semiconductor, Oslo, Norway)	nRF51422 (Nordic Semiconductor, Oslo, Norway)
**Center Frequency**	2400 MHz	
**Transmission Power**	+4 dBm	-
**Receiver Sensitivity**	-	−85 dBm
**Bandwidth**	2 Mbps	
**Antenna Type**	ANT-2.45-CHP-T (Linx Technologies Inc., Merlin, OR, USA) SMD	Quarter-Wavelength Monopole PCB
**Sample Rate**	1 ksps	

**Table 2:** Parameters of the path loss model shown in Equations (1) and (2) for all studied technologies.

		450 MHz Monopole	450 MHz BCC	915 MHz Parallel	915 MHz Orthogonal	2 GHz Monopoles	2 GHz Antenna Arrays	2.4 GHz Bluetooth Nodes
**Model 1**	***P*_0_ (dB)**	−21	−52	−44	−14	−19	−25	−62
	***n***	3.2	1.8	0.57	2.5	2.0	2.4	1.7
	$\sigma_{p}$ **(dB)**	2.8	6.2	2.6	1.9	1.3	1.3	1.8
**Model 2**	***P*_0_ (dB)**	−19	−51	−44	−13	−17	−24	−62
	$m_{0}$ **(dB/cm)**	0.52	0.30	0.087	0.38	0.34	0.39	0.23
	$\sigma_{p}$ **(dB)**	3.6	6.2	2.6	1.8	1.1	1.9	2.4

**Table 3:** Parameters of the path loss model shown in Equations (1) and (2) for five scenarios and two studied antenna configurations at 915 MHz.

		Antenna Parallel					Antenna Parallel				
		Around Torso	Back	Chest	Arm	Leg	Around Torso	Back	Chest	Arm	Leg
**Model 1**	***P*_0_ (dB)**	−30	−23	−33	−44	−40	−9.7	−19	−16	−14	−14
	***n***	5.0	3.4	2.9	0.57	1.6	5.3	2.9	4.2	2.5	2.3
	$\sigma_{p}$ **(dB)**	4.1	3.6	3.3	2.6	4.5	4.8	2.7	3.3	1.9	4.3
**Model 2**	***P*_0_ (dB)**	−29	−21	−29	−44	−43	−6.6	−17	−12	−13	−16
	$m_{0}$ **(dB/cm)**	0.75	0.54	0.57	0.087	0.14	0.85	0.45	0.72	0.38	0.24
	$\sigma_{p}$ **(dB)**	5.7	3.5	3.2	2.6	5.2	4.7	2.2	3.0	1.8	4.2
